# Diversity and Evolution of Integrative and Conjugative Elements Involved in Bacterial Aromatic Compound Degradation and Their Utility in Environmental Remediation

**DOI:** 10.3390/microorganisms11020438

**Published:** 2023-02-09

**Authors:** Jun Hirose

**Affiliations:** Department of Applied Chemistry, Faculty of Engineering, University of Miyazaki, Miyazaki 889-2192, Japan; jhirose@cc.miyazaki-u.ac.jp; Tel.: +81-985-58-7322

**Keywords:** aromatic compound, bioremediation, genomic island, integrative and conjugative element, horizontal gene transfer

## Abstract

Integrative and conjugative elements (ICEs) are mobile DNA molecules that can be transferred through excision, conjugation, and integration into chromosomes. They contribute to the horizontal transfer of genomic islands across bacterial species. ICEs carrying genes encoding aromatic compound degradation pathways are of interest because of their contribution to environmental remediation. Recent advances in DNA sequencing technology have increased the number of newly discovered ICEs in bacterial genomes and have enabled comparative analysis of their evolution. The two different families of ICEs carry various aromatic compound degradation pathway genes. ICE*clc* and its related ICEs contain a number of members with diverse catabolic capabilities. In addition, the Tn*4371* family, which includes ICEs that carry the chlorinated biphenyl catabolic pathway, has been identified. It is apparent that they underwent evolution through the acquisition, deletion, or exchange of modules to adapt to an environmental niche. ICEs have the property of both stability and mobility in the chromosome. Perspectives on the use of ICEs in environmental remediation are also discussed.

## 1. Introduction

Bacteria can adapt to environmental niches by acquiring preexisting phenotypes from bacterial genetic resources. This is accomplished by the coordinated activities of mobile genetic elements, including insertion sequences, transposons, integrons, other cell-to-cell transmissible plasmids, and chromosomally integrated mobile genetic elements (ICEs) that transfer within or between DNA molecules [1]. ICEs are genetic elements consisting of several tens to 200 kb of DNA that carry genes involved in drug resistance, pathogenicity, metabolism, and symbiosis with plants. They contribute considerably to the genomic evolution of environmental microorganisms because they are horizontally transfered via conjugation across bacterial species. Although many ICEs involved in drug resistance and virulence have been identified in clinically isolated bacteria [2], there are limited reports on ICEs containing other functional genes [3,4]. A group of ICEs, that contain cargo genes involved in the degradation of aromatic compounds, can contribute to environmental remediation. Some genes encoding aromatic degradation pathways are chromosomally encoded, whereas some are encoded by plasmids. For example, the *bph* genes encoding the biphenyl and chlorobiphenyl *meta*-cleavage degradation pathways in *Cupriavidus oxalacticus* A5 (formerly *Alcaligenes eutrophus* A5) [5], *Acidovorax* sp. KKS102 [6], and various *Pseudomonas* species [7] are located on the chromosome, whereas multiple *bph* genes from the Gram-positive bacterium *Rhodococcus jostii* RHA1 are located on a large linear plasmid [8]. Genome sequence analysis has revealed that a larger part of chromosomally encoded *bph* genes are located on ICEs.

‘Degradative ICEs’ comprise a set of genes that encode enzymes that are essential for the catabolism of various toxic chemicals. These degradative ICEs are large mobile genetic elements that contain a complete set of genes encoding components necessary for chromosomal excision, integration and conjugative transmission with the donor strain [3,4]. ICEs can be transferred from one strain to another via horizontal gene transfer (HGT); thus, ICE-encoded catabolic pathways enable the transfer of specific catabolic genes in microbial populations, thereby enabling microbial adaptation to toxic organic pollutants in the environment. The transmission of catabolic genes between communities helps bacterial strains to survive in environmental niches and plays an important role in the evolution of catabolic pathways. Although many ICEs have also been found in several Gram-positive bacteria [3], ICEs involved in the degradation of aromatic compounds are rarely found from the Gram-positive bacteria. Among the degradative ICEs responsible for the degradation of aromatic compounds, the ”ICE*clc* family”, which includes ICE*clc*B13 [9], the most well-studied degradative ICE, includes ICEs involved in most degradation pathways reported to date. The “Tn*4371* family” comprises a group encompassing several ICEs that exhibit relatively broad host specificity and carry genes involved in chlorinated-biphenyl degradation [6,10]. A *phn* island that encodes the phenanthrene catabolic pathway as a cargo gene contains a core region belonging to an unidentified family [11]. 

With recent advances in genome sequencing technology, new ICEs are being discovered [12,13]. Recently, several ICE*_bph-sal_*s and ICE*_bph_*s have been identified from the genome sequences of biphenyl/PCB -degrading bacteria, and their entire structures have been elucidated [7]. Another new degradative ICE, ICE*nah*CSV86 from a well-characterized aromatic degrader, *Pseudomonas bharatica*, was also reported [14]. It is becoming clear that ICEs are diversifying through the acquisition and rearrangement of cargo genes and contributing to environmental remediation. This article reviews the structure, functions and the evolution of previously reported ICEs involved in aromatic degradation.

## 2. Metabolic Pathway Genes Transported by ICEs

### 2.1. Aromatic Hydrocarbons

**Figure 1:**
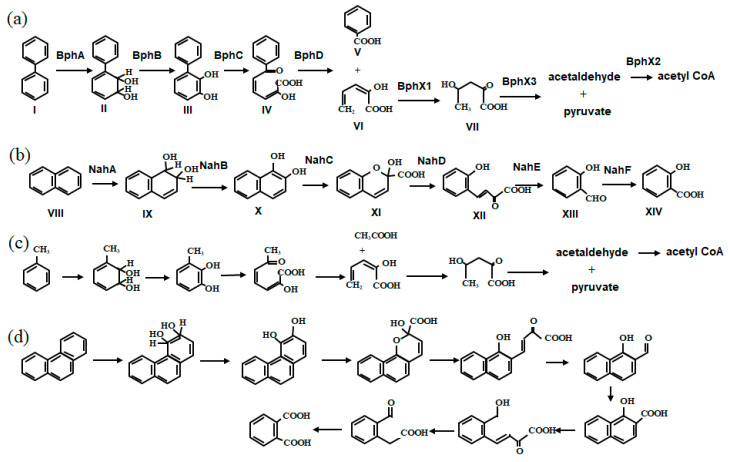
Aerobic degradation pathways for aromatic hydrocarbons encoded on degradative ICEs: (**a**) biphenyl, (**b**) naphthalene, (**c**) toluene, and (**d**) phenanthrene. The names of the intermediate compounds are as follows: biphenyl (I), *cis*-2,3-dihydroxy-4-phenylhexa-4,6-diene (dihydrodiol compound, II), 2,3-dihydroxybiphenyl (III), 2-hydroxy-6-oxo-6-phenylhexa-2,4-dienoic acid (IV), benzoic aid (V), 2-hydroxypenta-2,4-dienoic acid (VI), 4-hydoxy-2-oxovaleric acid (VII), naphthalene (VIII), *cis*-1,2-dihydroxy-1,2-dihydronaphthalene (IX), 1,2-dihydroxynaphthalene (X), 2-hydroxychromene-2-carboxylate (XI), *o*-hydroxybenzylidenepyruvate (XII), salicylaldehyde (XIII), and salicylic acid (XIV). Enzyme names on each arrow are as follows: BphA, biphenyl 2,3-dioxygenase; BphB, 2,3-dihydro-2,3-dihydroxybiphenyl dehydrogenase; BphC, 2,3-dihydroxybiphenyl 1,2-dioxygenase; BphD, 2-hydroxy-6-oxo-6-phenylhexa-2,4-dienoate hydrolase; BphX1, 2-hydroxypenta-2,4-dienoate hydratase; BphX2, acetaldehyde dehydrogenase; BphX3, 4-hydoxy-2-oxovalerate aldolase; NahA, naphthalene 1,2-dioxygenase; NahB, 1,2-dihydroxy-1,2-dihydronaphthalene dehydrogenase; NahC, 1,2-dihydroxynaphthalene dioxygenase; NahD, 2-hydroxychromene-2-carboxylate isomerase; NahE, *trans*-*o*-hydroxybenzylidenepyruvate hydratase-aldolase; and NahF, salicylaldehyde dehydrogenase. The intermediate compound names and enzyme names in (**c**,**d**) are described in references [17] and [11], respectively.

Many aromatic hydrocarbon-utilizing bacteria have been isolated and characterized. The ICEs identified in their genomes are involved in the dissimilation of biphenyl (Figure 1a), naphthalene (Figure 1b), toluene (Figure 1c) and phenanthrene (Figure 1d), which are aromatic hydrocarbons in petroleum and coal. The metabolism of aromatic compounds by degradation pathway genes, including those located in ICEs, generally consists of two processes: (1) introduction of a hydroxyl group to an aromatic ring, and (2) cleavage of a hydroxylated aromatic ring [15].

1. Aromatic ring hydroxylation: Hydroxylation of the aromatic ring is catalyzed by a dioxygenase that introduces two oxygen atoms into the ring or by a monooxygenase that introduces one oxygen atom. The former introduces two hydroxyl groups into an aromatic ring, whereas the latter introduces one hydroxyl group into an aromatic ring or a side-chain alyl-group. Both dioxygenase and monooxygenase encoded by genes in ICEs are multi-component enzymes [16].
2. Aromatic ring cleavage: The cleavage of the aromatic ring can be grouped into two modes. The first is *meta*-cleavage, in which the carbon-carbon bond between the 2- and 3-positions of the catechol is cleaved by the addition of molecular oxygen, producing an intermediate that is generally yellow. The second is *ortho*-cleavage, in which the bond between the two hydroxyl groups is cleaved to produce a colorless intermediate [16].

### 2.2. Aromatic Carboxylic Acids

**Figure 2:**
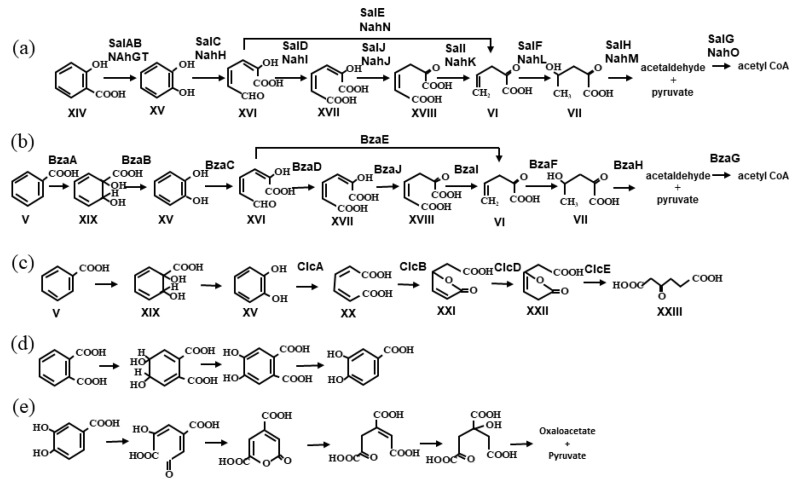
Aerobic degradation pathways for aromatic carboxylic acids (**a**) salicyliate (**b**) benzoate (*meta*-pathway), (**c**) benzoate (*ortho*-pathway), (**d**) phthalate and (**e**) protocatechuate encoded on degradative ICEs. The names of the intermediate compounds are as follows: salicylic acid (XIV), catechol (XV), 2-hydroxy-*cis,cis*-muconate semialdehyde (XVI), 2-hydroxy-hexa-2,4-diene-1,6-dioic acid (XVII), 2-oxohex-4-ene-1,6-dioic acid (4-oxalocrotonate) (XVIII), *cis*-1,6-dihydroxy-2,4-cyclohexadiene-1-carboxylic acid (XIX), *cis,cis*-muconate (XX), muconolactone (XXI), 3-oxoadipate enol-lactone (XXII), and 3-oxoadipic acid (XXIII). The compound names of (V), (VI), and (VII) are same as those in Figure 1. The Enzyme names on each arrow are: SalAB/NahGT, salicylate hydroxylase; BzaA, benzoate 1,2-dioxygenase; BzaB, 2-hydro-1,2-dihydroxybenzoate dehydrogenase (benzoate *cis*-diol dehydrogenase); SalC/NahH/BzaC, catechol 2,3-dioxygenase; SalD/NahI/BzaD, hydroxymuconic semialdehyde dehydrogenase; SalE/NahN/BzaE, 2-hydroxymuconate semialdehyde hydrolase; SalJ/NahJ/BzaJ, 4-oxalocrotonate isomerase; SalI/NahK/BzaI, 4-oxalocrotonate decarboxylase; SalF/ NahL,/BzaF, 2-oxopent-4-enoate hydratase; SalH/NahM/ BzaH, 2-oxo-4-hydroxypentanoate aldolase; SalG/NahO/BzaG, acetaldehyde dehydrogenase; ClcA, catechol 1,2-dioxygenase; ClcB, muconate cycloisomerase; ClcD, muconolactone D-isomerase; and ClcE, 3-oxoadipate enol-lactonase. The intermediate compounds names and enzyme names in (**d**,**e**) are described in reference [11].

Bacteria that utilize aromatic carboxylic acids such as benzoic acid and salicylic acid are widely distributed in nature. Some of the genes involved in the degradation of aromatic carboxylic acids are cargo genes in ICEs. Benzoic acid is a metabolic intermediate of biphenyl (Figure 1a), whereas salicylic acid is an intermediate of the naphthalene metabolic pathway (Figure 1b). In addition, phthalates are intermediates of phenanthrene degradation (Figure 1d) [11]. Aromatic compounds undergo cleavage reactions to become aliphatic products, which then enter the tricarboxylic acid cycle through reactions such as hydrolysis, decarboxylation, isomerization, and aldol cleavage to acetyl-CoA and pyruvate (Figure 2). The intermediates generated via *meta*-cleavage of catechol usually follow two branched pathways (Figure 2a,b). One pathway via oxidation of a ring-cleavage product to oxalocrotonate is a possible pathway for the degradation of *p*-toluic acid, an intermediate of *p*-xylene degradation, whereas the other short-circuit pathway via hydrolysis of a ring-cleavage product is dedicated to the dissmilation of *m*-toluic acid, an intermediate of *m*-xylene [18]. It is also known that an intermediate via the *meta*-cleavage pathway of monochlorobenzoic acid forms a dead-end intermediate from chlorocatechol [19]. This branched *meta*-cleavage pathway of catechol is commonly encoded by several ICE*clc* family members. In constrast, monochlorobenzoic acid can be completely degraded through the *ortho*-cleavage pathway to β-ketoadipic acid, especially in bacterial strains carrying ICE*clc*B13 (Figure 2c). Protocatechuic acid (3,4-dihydroxybenzoic acid) (Figure 2e) is a common metabolic intermediate of phthalic acid (Figure 2d), isoeugenol, ferulic acid, and vanillic acid [20]. The catabolic pathway via protocatechuic acid has not been reported to involve enzymes encoded by an ICE, except for phthalic acid pathway in the *phn* island [11].

### 2.3. Halogenated Aromatic Compounds

Halogenated hydrocarbons such as PCBs are degraded by the co-metabolism of bacteria possessing the degradadative ICEs described above. This process involves dehalogenation reactions during degradation. In particular, ICE, which has the *bph* gene encoding the biphenyl degradation pathway, plays an important role in the degradation of polychlorinated biphenyls (PCBs) in the environment [6,7,10]. The less-chlorinated homologs of PCB are converted to chlorobenzoic acids, whereas heavily chlorinated congeners produce dead-end intermediates [21]. Several ICEs have been discovered to carry the PCB degradation pathway as a cargo gene [6,7]. In contrast, 3-chlorobenzoic acid is mineralized by a series of chlorocatecholytic enzymes encoded by ICE*clc* [22].

## 3. Components Required for Conjugal Transfer of Degradative ICEs

### 3.1. Integrase

ICEs exist in two distinct states: an integrated state in which the DNA is located on the host chromosome, and a conjugative state in which the DNA is excised from the host chromosome and can potentially conjugate to a new cell. Integration results from site-specific recombination between two direct repeats that are a part of the attachment site (*att*), *attB* of the host chromosome and *attP* of the circular ICE. The integration reaction is catalyzed by integrase, and site-specific recombination results in the formation of the direct repeats *attL* (left end) and *attR* (right end) (usually 8–60 bp each) at both ends of the integrated ICE. The reverse reaction (excision) is also a site-specific recombination, leading to the integrated ICE’s release as closed circular DNA and putative repair of the chromosomal attachment site between the conserved repeats within *attL* and *attR*. Integrase is an essential component of both the integration and excision reactions. Known ICE integrases include tyrosine recombinase, serine recombinase and DDO transposase, whereas the ICE*clc* and Tn*4371* family members, which include degradative ICEs, possess tyrosine recombinase [3].

### 3.2. Transmission Module

The key components involved in conjugative transfer of degradative ICEs do not differ between ICEs and other mobile genetic elements such as transmissible plasmids. The transfer module is necessary for the transfer of an ICE from a donor to a recipient that forms the type IV secretion system (T4SS). It consists of a relaxase (MOB) and mating pair formation system (MPF)-coupled protein. MOB binds to the *oriT* of the circular intermediate of ICE to cleave one strand and binds to the 5’ end to pass through the MPF, a channel between the recipient and donor, leading to the transfer of single-stranded ICEs. Several MOB and MPF family members have been found in ICEs and transmissible plasmids, with different combinations of MOB and MPF between the ICE*clc* and Tn*4371* families [3].

### 3.3. Integration Site

ICEs transferred from a donor to a recipient bacterium form a circular intermediate that is integrated into the chromosome by integrase. The targets of ICE integration often differ among ICEs; they are located at the 5’ or 3’ ends of tRNA genes or other genes that encode housekeeping proteins. The integration sites of the ICE*clc* and Tn*4371* family members are the 3’ ends of the tRNA-Gly and tRNA-Leu genes, respectively [3]. The integration of ICE into the target *attB* sequence results in the formation of 8 to 60 nucleotide direct repeat sequences, *attL* and *attR*. In addition, ICEs of the same family may show specificity for integration into tRNA-Gly containing different anticodons (e.g., tRNA-Gly-CCC and tRNA-Gly-GCC) [7]. ICEs belonging to the Tn*4371* family can be integrated into sites other than tRNA genes in certain hosts [6].

## 4. ICE*clc* Family

### 4.1. ICEclcB13

ICE*clc*B13 carries genes involved in the chlorobenzoic acid and aminophenol degradation pathways as cargo genes. It was first discovered as a *clc* element present on the chromosome of *Pseudomonas knackmussii* B13 [9,22] that confers the ability to degrade chlorobenzoic acid. The presence of a mobile degradative element that uses a phage-like integrase was identified and considered the first degradative ICE [22]. ICE*clc*B13 transposes to various hosts belonging to Betaproteobacteria and Gammaproteobacteria, including *Pseudomonas* and *Cupriavidus* spp. [23,24,25]. Sequencing has revealed the overall structure of ICE*clc*B13 [26]: ICE integration sites are in the tRNA-Gly, integrase (tyrosine recombinase), 2-aminophenol catabolic gene cluster (*amnBACDFEHG*) and chlorocatechol catabolic gene cluster (*clcRABCDE*). The T4SS (VirB4, and VirD4) required for conjugative transfer of ICE, anthranilate 1,2-dioxygenase, drug efflux pumps, and putative DNA helicase genes have also been detected [26]. ICE*clc*B13 is excised through site-specific recombination of two 18-bp direct repeat sequences (*attL* and *attR*; 5′-GTCTCGTTTCCCGCTCCA-3′) flanking the integrated form, and then integrated into the chromosome [26].

*P. knackmussii* B13, the source of ICE*clc*B13, was originally discovered as a bacterium that can grow on 3-chlorobenzoic acid and 4-chlorobenzoic acid as its sole carbon sources [27]. 3- and 4-chlorobenzoic acids are converted to 3- or 4-chlorocatechol, respectively, by benzene or toluate dioxygenase and the subsequent dihydrodiol dihydrogenase, encoded in regions other than ICE*clc*B13, followed by conversion to β-adipate by the *ortho*-cleavage pathway encoded by the *clc* locus on ICE*clc*B13 [26]. Most of the aromatic ring-catabolic enzymes encoded by the cargo genes of degradative ICEs involve the *meta*-cleavage pathway, but ICE*clc*B13 is exceptional in that it encodes catabolic enzymes involved in the *ortho*-cleavage pathway and favors the degradation of chlorocatechol. ICE*clc*B13 contains a 2-aminophenol degradation gene involved in a pathway common to the *meta*-cleavage degradation of benzoic acid. ICE*clc*-carrying *Pseudomonas aeruginosa* uses 2-aminophenol as a carbon source, whereas *P. knackmussii* B13, the source of ICE*clc*B13, does not grow on 2-aminophenol [26]. This may result from metabolic pathway misroutes or the formation of toxic intermediates.

A remarkable feature of ICE*clc*B13 is its high transfer frequency from the donor to recipient cells. The transfer efficiency per donor cell reaches 1 × 10^−2^. ICE*clc*B13 can form multiple copies within the same chromosome [25,28]. InrR [29] and MfsR and TciR [30] have been identified as transcriptional regulators that govern the transfer frequency of ICE*clc*. Among these, MfsR has been suggested as a factor responsible for the highly efficient transfer of ICE*clc*B13 [4,30]. ICE*clc*B13 has two replication origins, *oriT*, both of which are known to be functional. The presence of duplicated *oriT*s appears to contribute to the high transfer frequency [31]. ICE*clc*B13 provides a good experimental model for investigating the mechanism of conjugative transfer of ICEs owing to its high transfer efficiency. By exploiting this property of ICE*clc*B13, the regulatory network of ICE*clc*B13 has been elucidated; ICE*clc*B13 is activated only in stationary-phase cells where activation is dependent on TciR transcriptional activator, and TciR stimulates unknown bistability generators, whose activation promotes the excision and transfer of ICE*clc*B13 [4,30].

### 4.2. ICEclcJB2 and ICEclcLB400

ICE*clc*JB2 [32] and ICE*clc*LB400 [26,33] are subspecies of ICE*clc* discovered by genomic sequence analysis of the *o*-chlorobenzoate-degrading bacterium *P*. *aeruginosa* JB2 and PCB-degrading bacterium *Paraburkholderia xenovorans* LB400 (formerly *Burkholderia xenovorans* LB400), respectively. ICE*clc*JB2 and ICE*clc*LB400 have almost the same core region as ICE*clc*B13 but have a unique structure of genes involved in the degradation of aromatic compounds that constitute their variable regions. ICE*clc*JB2 lacks *amn* gene cluster involved in aminophenol catabolism that is found on ICE*clc*B13 and ICE*clc*LB400, but instead possesses a duplicated *hyb* gene cluster required for salicylic acid utilization [34], which is absent in ICE*clc*B13 and ICE*clc*LB400 [32]. Additionally, ICE*clc*JB2 lacks an operon of regulatory genes (*tciR-marR-mfsR*) that is present in the other two ICE*clc* and which controls excision from the host [32]. These divergences in ICE*clc* are consistent with rearrangements through the acquisition, deletion, and duplication of aromatic compound gene modules.

### 4.3. ICE_XTD_

ICE*_XTD_* is an ICE derived from *Azoarcus* sp. CIB, a bacterium that can degrade *m*-xylene, toluene and cumene both anaerobically and aerobically and has a core region shared by ICE*clc* [17]. In addition to the ortholog of the aerobic toluene-degrading *tod* gene from *Pseudomonas putida* F1, ICE*_XTD_* possesses *bss* and *bzd* genes encoding the toluene-degrading pathway enzymes, which are often found in bacteria that degrade aromatic compounds anaerobically. The metabolic pathway involving enzymes encoded by the *tod* gene cluster is initiated by a reaction catalyzed by toluene dioxygenase (Figure 1c) and is distinct from that initiated by toluene monooxygenation of the side chain of toluene which is affiliated with the toluene catabolic plasmid pWW0. The reaction is followed by an attack by dioxygenase and *meta*-cleavage. The *meta*-cleavage compounds are degraded to TCA cycle intermediates through a metabolic pathway common to the downstream aerobic degradation pathways of biphenyl (Figure 1a). In addition, the toluene degradation pathway involving enzymes encoded by the *bss* and *bzd* gene clusters is a metabolic pathway initiated by the coupling of toluene with CoA via β-oxidation to produce acetyl-CoA [17]. A remarkable feature of this ICE is that it contains genes for both aerobic and anaerobic toluene degradation pathways. The transfer of ICE*_XTD_* confers *Cupriavidus pinatubonensis*, which originally does not degrade aromatic compounds under anaerobic conditions, with the ability to grow on *m*-xylene under anaerobic conditions [17]. The circular form of ICE*_XTD_* was formed by the recombination between two 23-bp direct repeat sequences (*attL* and *attR*; 5′-TTCGATTCCCATCGCCCGCTCCA -3′) [17]. The conjugative transfer frequency of ICE*_XTD_* from *Azoarcus* sp. CIB to *C. pinatubonensis* is 4.8 × 10^−7^ per donor strain. These results suggest that ICE are key elements for the survival of anaerobic bacteria in polluted environments.

### 4.4. ICE_bph-sal_

**Figure 3:**
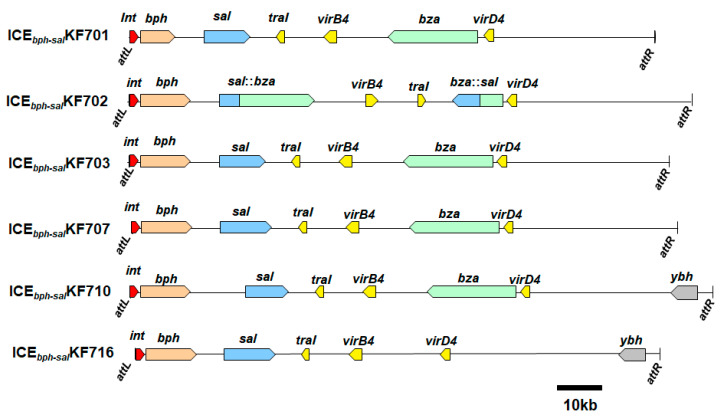
Organization of the ICE*_bph-sal_*s [7]. Genes on each arrow encode: *attL* and *attR*, ICE integration sites; *int*, integrase; *bph*, proteins involved in biphenyl catabolism; *sal*, proteins involved in salycilate catabolism*; traI*, relaxase (MOB); *virB4*, TSS4 component VirB4; *bza*, proteins involved in benzoate catabolism; *virD4*, TSS4 component VirD4; and *ybh*, YbhFSR ABC-type transporter.

Several ICE*_bph-sal_*, which are regarded as members of a subfamily of ICE*clc* that share a core region with ICE*clc*, harbor the *bph-sal* gene cluster that encodes enzymes of the degradation pathways via the *meta*-cleavage of biphenyl and salicylic acid [7,35]. It consists of the *bph* gene cluster *bphRA1A2A3A4BCX0X1X2X3D* and *sal* gene cluster *salABCDEFGHIJ* in ICE*_bph-sal_* [7]. In the biphenyl catabolic pathway encoded by the *bph* gene, biphenyl dioxygenase, a multicomponent enzyme encoded by *bphA1A2A3A4*, catalyzes the initial oxygenation of biphenyl and converts it to dihydrodiol (Figure 1a). Here, *bphA1* and *bphA2* encode the large and small subunits of terminal dioxygenase, respectively. *bphA3* encodes ferredoxin, and *bphA4* encodes ferredoxin reductase. The dihydrodiol compound is then converted to a dihydroxy compound by dehydrogenase encoded by *bphB*. The dihydroxy compound is then cleaved into 2-hydroxy-6-oxo-6-phenylhexa-2,4-dienoic acid by a ring-cleavage dioxygenase encoded by *bphC.* The ring *meta*-cleavage product is then cleaved into benzoic acid and 2-hydroxypenta-2,4-dienoic acid by a hydrolase encoded by *bphD*. BphX1, X2 and X3 further degrade 2-hydroxypenta-2,4-dienoic acid into acetyl-CoA and pyruvate. In the salicylate carabolic pathway encoded by the *sal* gene, salicylate hydroxylase, a binary enzyme encoded by *salAB*, catalyzes the hydroxylation of salicylate and converts it to catechol (Figure 2a). Catechol is then degraded to 2-hydroxymuconate semialdehyde by a ring-cleavage dioxygenase encoded by *salC*. The ring *meta*-cleavage product is then catabolized through two branching pathways. One ring-cleavage product is degraded to 2-hydroxypenta-2,4-dienoic acid via oxidation to oxalocrotonate by oxidase SalD and isomerization by SalJ. In contrast, the ring-cleavage product is hydrolyzed by SalE and converted directly to 2-hydroxypenta-2,4-dienoic acid. SalG, SalH, and SalF further degrade 2-hydroxypenta-2,4-dienoic acid to acetyl CoA and pyruvate. These structural *bph* and *sal* genes are coordinately regulated by *bphR* located in the *bph* gene cluster and *salR* located in the *sal* gene cluster [36]. Highly conserved ICE*_bph-sal_*s (ICE*_bph-sal_*KF701, ICE*_bph-sal_*KF702, ICE*_bph-sal_*KF703, ICE*_bph-sal_*KF707, ICE*_bph-sal_*KF710, and ICE*_bph-sal_*KF716) have been found in the genomes of six different biphenyl/PCB degrading *Pseudomonas* spp., for which whole-genome sequencing has been performed. Among these, ICE*_bph-sal_*KF716, which is located in the chromosome of *Pseudomonas stutzeri* KF716, forms a circular intermediate excised from the strain that is transferred to *P. aeruginosa* at a relatively high frequency [35]. ICE*_bph-sal_*KF716 is excised by site-specific recombination of two 18-bp direct repeat sequences (*attL* and *attR*; 5′-TTCCCTTCGCCCGCTCCA-3′) at both ends of the integrated form, and then integrated into the chromosome in the same manner as ICE*clc*B13 [26]. This direct repeat sequence differs by two bases between ICE*_bph-sal_* and ICE*clc*. The *attB* site, which is the closed form of the chromosome excised from the ICE, and the *attP* site, which is formed by linking the *attL* and *attR* sites of the excised ICE, are generated [35]. All six ICE*_bph-sal_*s were found only in *Pseudomonas* spp., likely reflecting the strict host specificity of conjugative transfer. This is in contrast to the relatively broad host specificity of Tn*4371* and ICE_KKS102_*4677*, which are other biphenyl-degrading ICEs described below [6,10]. The overall structure of ICE*_bph-sal_* is well conserved, but parts of the structures are different (Figure 3). ICE*_bph-sal_*s except for ICE*_bph-sal_*KF716 has a *bzaABCDEFGHIJ* gene cluster encoding enzymes of the benzoate *meta*-cleavage pathway (Figure 2b). They have a homolog of the lower *xyl* operon on the toluene degradative plasmid pWW0 [37], and the carboxylic acid transporter that is not present in pWW0. In contrast, ICE*_bph-sal_*KF716 lacks the *bza* cluster. In addition, ICE*_bph-sal_*KF710 and ICE*_bph-sal_*KF716 have *ybh* gene cluster, an orthologue of the *Escherichia coli* YbhFSR ABC-type efflux transporter [38], whereas other ICE*_bph-sal_*s do not (Figure 3). ICE*_bph-sal_*s, except for ICE*_bph-sal_*KF716, have three sets of aromatic ring *meta*-cleavage pathway genes: biphenyl (*bph*), salicylic acid (*sal*), and benzoic acid (*bza*). Most of them are duplicated or triplicated (Figure 1a, and 2ab) [7]. ICE*_bph-sal_*KF702 undergoes partial structural inversion owing to recombination between homologous genes duplicated between *sal* and *bza*. These findings indicate that ICE*_bph-sal_*s rearrange through cargo gene module acquisition, exchange, and recombination [7]. A degradative ICE containing a highly conserved core region almost identical to that of ICE*_bph-sal_*KF716 has been found in silico suggesting the existence of an ”ICE*_bph-sal_* subfamily”; they include ICE members located on the chromosome of naphthalene-degrading bacterium *P. stutzeri* AN10 and toluene/xylene and phenol-degrading bacterium *P. stutzeri* 2A20 [35]. All of these ICEs have been found in *P. stutzeri*, suggesting that *P. stutzeri* is the preferred host of the ICE*_bph-sal_* subfamily [39].

A mobile element carrying a conserved *bph-sal* cluster identical to that of ICE*_bph-sal_*s is the large IncP-9 family plasmid pKF715A (483-kb) derived from *P. putida* KF715 [40]. The major part of pKF715A behaves like ICE, being chromosomally integrated into host cells, and a mixture of circular and chromosomally integrated plasmids has been detected in *P. putida* KF715. pKF715A is transferred from *P. putida* KF715 to *P. putida* F1 and integrated into the chromosome [7]. pKF715A contains an integrase homologous to that of ICE*_bph-sal_*s as well as a replication gene (*rep*), an origin of transfer (*oriT*) sequence, a plasmid conjugal transfer gene (*tra*), and a plasmid partitioning gene (*par*) homologous to those of the toluene catabolic plasmid pWW0.

### 4.5. ICEnahCSV86

ICE*nah*CSV86 from *P. bharatica* CSV86 was recently reported to be an ICE*clc* family member [14]. ICE*nah*CSV86 carries the *nah* gene encoding the naphthalene degradation pathway and the *sal* gene encoding the salicylate *meta*-cleavage degradation pathway, corresponding to the downstream naphthalene degradation pathway. The *nah* gene cluster on ICE*nah*CSV86 shows common structure with the *nah* gene cluster on the naphthalene degradative plasmid NAH7, consisting of the upper *nah* gene (*nahAaAbAcAdBFCED*) and lower *nah* gene (*nahRGTHINLOMKJX*) [14]. The upper *nah* gene is responsible for the conversion of naphthalene to salicylic acid (Figure 1b). The *nahRGTHINLOMKJX* cluster, which encodes the lower *nah* genes responsible for the dissimilation of salicylic acid to pyruvate and acetyl-CoA, exhibits almost the same sequence and gene organization as the *sal* gene cluster included in ICE*_bph-sal_*s (Figure 2a). A transcriptional regulator encoded by *nahR*, located between the upper and lower *nah* gene clusters, regulates both the upper and lower *nah* genes [14]. An ICE of length approximately 100 kb, which is thought to be involved in naphthalene degradation and with a conserved structure homologous to that of ICE*nah*CSV86, has been found in the genomes of six other naphthalene-degrading bacteria [14], suggesting that ICEs or genomic islands similar to ICE*nah*CSV86 are widely distributed in the environment. The *sal* gene and most of the core region were almost identical between these ICEs carrying naphthalene catabolic *nah* genes and ICE*_bph-sal_*. Therefore, they can be considered members of the ICE*_bph-sal_* subfamily. After repeated growth, the unselective pressure of the ICE-encoded gene, ICE*nah*CSV86, was stably retained on the chromosome, together with the other two genomic islands, and did not affect naphthalene utilization [14]. This genetic stability appears to be a feature of ICE*nah*CSV86.

## 5. Tn*4371* Family

### 5.1. Tn4371

Tn*4371*, from *C. oxalaticus* A5, is an early discovered degradative ICE that is still referred to as a transposon. It satisfies the requirements of an ICE because it has genes involved in integration and genes involved in conjugal transfer. The latter genes display similarities to a conjugative gene on the Ti plasmid and IncP broad host range plasmid [10]. The 51-kb Tn*4371* was initially identified as IncP-1 plasmid pSS50, which is essential for the metabolism of 4-chlorobiphenyl to chlorobenzoic acid [41,42] but was later identified as a catabolic transposon that was inserted into the conjugative plasmid RP4 [5]. The RP4::Tn*4371* plasmid was maintained in different hosts, including *Acinetobacter, Chromobacterium,* and *Pseudomonas* species. It confers biphenyl/4-chlorobiphenyl degradability only to *C. oxalaticus* and *Acinetobacter* sp. but not to other species [43]. It was speculated that this results from the lack of regulatory elements or uptake systems. DNA sequencing revealed that Tn*4371* is a chromosomally integrated mobile genetic element with integrase (tyrosine recombinase), a T4SS common to IncP and Ti plasmids, and a gene cluster encoding biphenyl *meta*-cleavage enzymes [10]. Many putative ICEs that share a core region with Tn*4371* have been found in the genome sequences of at least nine different bacterial species belonging to Betaproteobacteria and ten different species belonging to Gammaproteobacteria [6,43,44], of which ICEs from *Burkholderia*, *Bordetella*, *Polaromonas* and *Stenotrophomonas* contained putative degradative genes for aromatic compounds [43]. They are thought to be widely distributed in the genomes of bacteria in the environment.

### 5.2. ICE_Tn4371_6054

*Cupriavidus metallidurans* CH34 harbors at least three Tn*4371* family members on its genome: ICE_Tn*4371*_6054, ICE_Tn*4371*_6055 and ICE_Tn*4371*_6056. ICE_Tn*4371*_6054 is the second reported Tn*4371* family member of degradative ICEs [45,46]. ICE_Tn*4371*_6054 contains genes encoding *meta*-cleavage-degrading enzymes including toluene monooxygenase as the intial oxygenase. It contains the hydrogenase gene adjacent to the catabolic cluster, as a cargo gene, which is presumed to be involved in the chemolithotrophic growth of this strain [47]. Information regarding the functions of this genetic element, such as its mobilization and catabolic capabilities is limited

### 5.3. ICE_KKS102_4677

ICE_KKS102_*4677* is a Tn*4371* family member ICE identified using genome sequence analysis of the PCB/biphenyl-degrading bacterium *Acidovorax* sp. strain KKS102 [6]. Each component of the biphenyl catabolic enzymes of the *bphSEGFVA1A2A3BCDWA4* gene cluster on ICE_KKS102_*4677* shares similarities with those of the *bphRA1A2A3A4BCX0X1X2X3D* gene cluster on ICE*_bph-sal_*; however, the gene organization and regulatory mechanisms differ significantly between the *bph* genes [6]. The *bph* gene in ICE_KKS102_*4677* is regulated by the transcriptional repressors BphS [48] and a two-component regulatory system of BphPQ [49]. ICE_KKS102_*4677* was transferred to a wide range of hosts, including *Pseudomonas*, *Burkholderia*, and *Sphingobium*, and integrated into the chromosome. Although the main target for chromosomal integration is the tRNA-Leu gene, it can integrate at different sites, depending on the host [6]. The ratio of circular intermediates of ICE_KKS102_*4677* excised from the chromosome to those integrated into the chromosome was reported to be 1 × 10^−5^. The transfer efficiency per donor cell of ICE_KKS102_*4677* from *Acidovorax* sp. KKS102 to *P. putida* KT2440 is reportedly 5.8 × 10^−10^, which is considerably lower than the transfer frequency of other ICEs [6]. The reason for the low transfer frequency of ICE_KKS102_*4677* remains unknown.

### 5.4. ICE_bph_KF708 and ICE_bph_KF712

ICE*_bph_*KF708 and ICE*_bph_*KF712 are 61.8 kb and 59.4 kb, respectively, and are Tn*4371* family members of the biphenyl/PCB-degrading bacteria *Cupriavidus basilensis* KF708 and *Comamonas testosteroni* KF712, respectively [7]. ICE*_bph_*KF708 is closely related to ICE_KKS102_*4677* and ICE*_bph_*KF712 to Tn*4371*; the ICE integration (the *att* sequence) site of ICE*_bph_*KF708 and ICE_KKS102_*4677* is 5’-GATTTTAAG-3’, and that of ICE*_bph_*KF712 and Tn*4371* is 5’-TTTTTCAT-3’. In addition, ICE*_bph_*KF708 and ICE_KKS102_*4677* carry a putative arsenic resistance gene cluster 15.5 to 18.5 kb downstream from the *attL* site, whereas ICE*_bph_*KF712 and Tn*4371* do not [7]. The sequence of the core region covering 40 to 43 kb of the total, including *bph* and *trb* genes of these Tn*4371*-type ICEs, is highly conserved, but 10 to 20 kb of the *attL* site, except for the *int* gene encoding integrase, is a so-called junk region that encodes many unidentified proteins, and its sequence is not conserved among ICE*_bph_*KF708, ICE*_bph_*KF712 and Tn*4371* [7].

## 6. ICE Belonging to an Unidentified Family

### phn Island

The *phn* island is derived from the phenanthrene-degrading bacterium *Delftia acidovorans* Cs1-4 [11,50] and shares no core regions with ICEs belonging to known families. It can be considered to be an ICE because the genome of *D. acidovorans* SPH1 does not contain a *phn* island and carries a group of components characteristic of an ICE, such as P4-type phage integrase and Tra proteins that constitute T4SS. The *phn* island internally encodes a LexA-like protein, a prophage repressor protein involved in the SOS response, and FlhC protein, a flagellar transcriptional activator of SXT/R391, an ICE family member found in *Vibriobacteriaceae* and *Enterobacteriaceae* [51], but their functions remain unclear. The *phn* includes (1) the *phn* cluster involved in the catabolism of phenanthrene to *o*-phthalate via the *meta*-cleavage pathway (Figure 1d), (2) the *oph* cluster involving the catabolism of *o*-phthalate to protocatechuate (Figure 2d), and (3) the *pmd* cluster, which is involved in the catabolism of protocatechuate to pyruvate and oxaloacetate via the *meta*-cleavage pathway (Figure 2e). The phenanthrene-converting function of *phn* has been confirmed with experiments using a *phn* deletion mutant and heterologous expression in *Escherichia coli*. The GC content of the *phn* gene cluster is significantly lower than that of other regions of the *phn* island, suggesting the exogenous nature of the *phn* gene cluster.

## 7. Evolution of Degradative ICEs

The degradative ICEs in the ICE*clc* family and Tn*4371* family are listed in Table 1 and Table 2, respectively. Between the ICE families, the accumulation of sequence data has enabled comparative analysis of the evolution of the ICE*clc* family members. Differences in the substructures of several degradation ICEs indicate that these genetic elements evolve through module exchange, insertion, and rearrangement to adapt to an environmental niche. Mobile elements such as IS and transposons contribute to the exchange of modules between ICEs. The multiple mobile genes encoding transposases and a retron encoding retron-type reverse transcriptases are inserted upstream and downstream of the *sal* gene in the *bph-sal* clusters of ICE*_bph-sal_*s [40]. It has been speculated that they contribute to the exchange of the *bph* gene with the upper *nah* operon, the counterpart of the *bph* gene in the *nah-sal* cluster which is involved in naphthalene catabolism on the ICE [35]. However, the mechanisms governing the module exchange between ICEs are largely unknown. It has been pointed out that gene exchange between ICEs and plasmids greatly contributes to the evolution of ICEs [3]. The *nah-sal* gene cluster of ICE*nah*CSV84 and *sal* gene cluster of ICE*_bph-sal_*s exhibit similarities with those of naphthalene degradation plasmids such as NAH7 and pND6-1 [14]. It has also been found that the *bza* gene cluster on ICE*_bph-sal_* is highly conserved with lower *xyl* genes on the TOL plasmid pWW0. The phylogenetic trees of VirB4, which constitute the T4SS encoded by the core region of ICE, were mixed with conjugative plasmids and ICE derivations at large phylogenetic distances [52]. This suggests an exchange of conjugative modules between plasmids and ICEs along the evolutionary history. Therefore, it can be concluded that the evolution of degradative ICEs and degradative plasmids is closely related in aromatic compound-degrading bacteria. Unlike plasmids, ICEs are integrated into the chromosome, and many microorganisms have multiple ICEs as genomic islands [14,28,32,33,45]. ICEs greatly contribute to genome evolution of aromatic compound-degrading bacteria. Further efforts on genome mining and functional analysis are expected to provide insights into the evolution of ICEs. 

Although both *ortho*- and *meta*-cleavage pathways function in many aromatic-degrading bacteria, the degradation pathways encoded on the previously discovered degradative ICEs are all *meta-*cleavage pathways, except for the *ortho*-cleavage pathway of chlorocatechol involving a gene on ICE*clc* [6,7,11,14,17,26]. The *meta*-cleavage pathway is disadvantageous because of the production of toxic aldehydes and dead-end products in the degradation of chlorocatechol as catabolic intermediates [19]. The question that needs to be addressed is why degradative ICEs are recruiting *meta*-cleavage pathways. One hypothesis is that the branching pathway from the ring-cleavage product (Figure 2a,b), which is commonly facilitated by the *meta*-cleavage pathways encoded on many degradative ICEs, enables the dissimilation of a wide range of substrates. It should also be noted that many degradable ICEs of the ICE*clc* family possess genes that encode multiple redundant *meta*-cleavage pathways for two or more different substrates, as shown in Table 1. For example, the biphenyl, salicylate, and benzoate metabolic enzymes encoded by the *bph*, *sal*, and *bza* gene clusters present on ICE*_bph-sal_*s catalyze overlapping reactions (Figure 1a and Figure 2a,b) [7]. The acquisition of such redundancy in cargo genes is common in the evolution of degradable ICEs.

## 8. Prospects for Use in Bioremediation

ICEs, which carry genes encoding aromatic compound degradation pathways, contribute to environmental remediation. In particular, ICEs which has *bph* gene that encodes the biphenyl degradation pathway, play an important role in the degradation of PCBs in the environment. In contaminated sites, bacteria with mobile genetic elements such as plasmids and ICEs can transfer mobile genetic elements to different strains. They help bacterial strains adapt to new environments and transform them to exhibit diverse phenotypes in response to selective pressure. This mechanism spreads catabolic genes to bacterial populations in contaminated sites [53]. Gunsch et al. proposed genetic (plasmid-mediated) bioaugmentation to introduce donor bacteria harboring self-transferable catabolic plasmids into the soil matrix to increase the likelihood and rate of contaminant degradation by pre-existing bacterial populations through HGT [54]. In cellular bioaugmentation, the inoculated strains are expected to degrade xenobiotics, whereas in genetic bioaugmentation, catabolic genes placed in mobile genetic elements are expected to be transferred to local microbial communities. Thus, one of the major limitations of cell bioaugmentation, the low viability of inoculated microorganisms, should be overcome via genetic bioaugmentation. Plasmid genes are susceptible to deletion and are unstable; however, ICEs are often stably maintained in the host without deletion. In addition, unlike indigenous genes on the chromosome, they also have mobility which makes heterologous transformation possible [6,23,25]. As ICEs do not exhibit plasmid-like incompatibility, they can be integrated multiple times into the chromosome [25,29]. In fact, many bacteria in the environment have multiple different ICEs on their chromosomes [14,28,32,33,45]. By exploiting such properties of ICEs, it is possible to enhance the degradation of pollutants by transducing multiple degradable ICEs with different aromatic compound-degrading properties. As ICEs occur naturally, heterologous transconjugants of ICEs obtained through conjugative transfer are essentially non-recombinant bacteria. Therefore, they can be applied to environmental remediation without the regulations applicable for genetically modified organisms. An early study bred chlorobiphenyl-degrading bacteria via the heterologous introduction of Tn*4371* into chlorobenzoate-degrading bacteria [55]. Using the latest bioinformatic, proteomic, and metabolomic methods, molecular breeding via heterologous introduction of ICEs can be achieved in a more sophisticated manner [24]. The introduction of ICE-carrying degrading bacteria in contaminated sites is expected to result in genetic bioaugmentation through HGT of degrading genes. The transfer of Mini-ICEBs1 in soil has been demonstrated [56]. From the above point of view, it seems necessary to control the stability and mobility of ICE to some extent if degradative ICEs are applied to environmental remediation. Disruption of the ICE*clc* transcriptional regulator has been reported to alter the mobility of ICE*clc*B13, but it has not yet been successfully controlled [29,30]. In the future, ICEs can be controlled by elucidating the role of all factors that control the mobility of ICEs. 

A problem with the remediation of contaminated sites with halogenated compounds such as PCBs is the difficulty in degrading heavily chlorinated compounds [21,57], but dechlorination by anaerobic reduction is possible to some extent. These compounds are reported to be easily degraded by a combination of anaerobic and aerobic processes [58,59]. ICE*_XTD_* carries genes involved in both aerobic and anaerobic aromatic hydrocarbon degradation pathways as cargo genes and has been confirmed to confer anaerobic toluene-degrading ability to other bacteria through conjugative transfer [17]. ICEs such as ICE*_XTD_* are likely to facilitate the remediation of such heavily chlorinated contaminants.

## 9. Conclusions

The degradative ICEs belonging to two families, Tn*4371* and ICE*clc*, have been listed. Undoubtedly, they are deeply involved in the HGT of aromatic compound degradation pathways in environmental bacteria. Among the ICE families, the accumulation of sequence data has enabled comparative analyses of the evolution of the ICE*clc* family members. It is clear that genes evolve in the environment through rearrangement and modification through acquisition, deletion and exchange of modules to adapt to environmental niches. Further elucidation of the structure and function of ICEs is expected to provide insights into the diversity and evolution of ICEs. In addition, environmental remediation that does not use recombinant microorganisms could be achieved by exploiting the characteristics of ICEs, which exhibit both stability and mobility in the chromosome.

## Figures and Tables

**Table 1:** ICE*clc* family members.

ICE	Size (kb)	Origin	Aromatic Compounds Targeted by Degradative Genes	References
ICE*clc*B13	102.8	*Pseudomonas knackmussii* B13	Aminophenol, chlorocatechol	[9,22,23,24,25,26,28,29,30,31]
ICE*clc*JB2	123.3	*Pseudomonas aeruginosa* JB2	*o*-Chlorobenzoate, salicylate	[32]
ICE*clc*LB400	122.8	*Paraburkholderia xenovorans* LB400	Aminophenol, chlorocatechol	[26,33]
ICE*_XTD_*	173.8	*Azoarcus* sp. CIB	Toluene, *m*-xylene	[17]
ICE*nah*CSV84	105.2	*Pseudomonas bharatica* CSV86	Naphthalene, salicylate	[14]
ICE*_bph-sal_*KF701	117.4	*Pseudomonas abietaniphila* KF701	Biphenyl, salicylate, benzoate	[7]
ICE*_bph-sal_*KF702	125.7	*Pseudomonas aeruginosa* KF702	Biphenyl, salicylate, benzoate	[7]
ICE*_bph-sal_*KF703	120.8	*Pseudomonas putida* KF703	Biphenyl, salicylate, benzoate	[7]
ICE*_bph-sal_*KF707	122.0	*Pseudomonas furukawaii* KF707	Biphenyl, salicylate, benzoate	[7]
ICE*_bph-sal_*KF710	130.3	*Pseudomonas toyotomiensis* KF710	Biphenyl, salicylate, benzoate	[7]
ICE*_bph-sal_*KF716	117.3	*Pseudomonas stutzeri* KF716	Biphenyl, salicylate	[7,35]

**Table 2:** Tn*4371* family degradative ICE members.

ICE	Size (kb)	Origin	Aromatic Compounds Targeted by Degradative Genes	References
Tn*4371*	54.7	*Cupriavidus oxalacticus* A5	Biphenyl	[5,10]
ICE_Tn*4371*_6054	101	*Cupriavidus metallidurans* CH34	Toluene	[43,44]
ICE_KKS102_4677	61.8	*Acidovorax* sp. strain KKS102	Biphenyl	[6]
ICE*_bph_*KF708	61.8	*Cupriavidus basilensis* KF708	Biphenyl	[7]
ICE*_bph_*KF712	59.4	*Comamonas testosteroni* KF712	Biphenyl	[7]
